# Electron Backscattered Diffraction to Estimate Residual Stress Levels of a Superalloy Produced by Laser Powder Bed Fusion and Subsequent Heat Treatments

**DOI:** 10.3390/ma13204643

**Published:** 2020-10-17

**Authors:** Mathieu Terner, Jiwon Lee, Giulio Marchese, Sara Biamino, Hyun-Uk Hong

**Affiliations:** 1 Department of Materials Science and Engineering, Changwon National University, 20 Changwondaehak-ro, Changwon, Gyeongnam 51140, Korea; jwlee10@changwon.ac.kr (J.L.); huhong@changwon.ac.kr (H.-U.H.); 2 Institut Clément Ader (ICA), Université de Toulouse, CNRS, IMT Mines Albi, INSA, ISAE-SUPAERO, UPS, Campus Jarlard, F-81013 Albi, France; 3 Department of Applied Science and Technology, Politecnico di Torino, Corso Duca degli Abruzzi 24, 10129 Torino, Italy; giulio.marchese@polito.it (G.M.); sara.biamino@polito.it (S.B.)

**Keywords:** laser powder bed fusion, superalloys, residual stress, heat treatment, electron backscattered diffraction, Alloy 625

## Abstract

Metal Additive Manufacturing and Laser Powder Bed Fusion (LPBF), in particular, have come forth in recent years as an outstanding innovative manufacturing approach. The LPBF process is notably characterized by very high solidification and cooling rates, as well as repeated abrupt heating and cooling cycles, which generate the build-up of anisotropic microstructure and residual stresses. Post-processing stress-relieving heat treatments at elevated temperatures are often required in order to release some of these stresses. The effects of 1 h–hold heat treatments at different specific temperatures (solutionizing, annealing, stress-relieve and low-temperature stress-relieve) on residual stress levels together with microstructure characterization were therefore investigated for the popular Alloy 625 produced by LPBF. The build-up of residual stress is accommodated by the formation of dislocations that produce local crystallographic misorientation within grains. Electron backscattered diffraction (EBSD) was used to investigate local misorientation by means of orientation imaging, thereby assessing misorientation or strain levels, in turn representing residual stress levels within the material. The heavily constrained as-built material was found to experience full recrystallization of equiaxed grains after solutionizing at 1150 °C, accompanied by significant drop of residual stress levels due to this grains reconfiguration. Heat treatments at lower temperatures however, even as high as the annealing temperature of 980 °C, were found to be insufficient to promote recrystallization though effective to some extent to release residual stress through apparently dislocations recovery. Average misorientation data obtained by EBSD were found valuable to evaluate qualitatively residual stress levels. The effects of the different heat treatments are discussed and suggest that the peculiar microstructure of alloys produced by LPBF can possibly be transformed to suit specific applications.

## 1. Introduction

For several years, Metal Additive Manufacturing has become an undisputable alternative to conventional manufacturing. It offers many advantages including versatility, complex and near-net-shape manufacturing, elimination of tooling, materials waste reduction, shorter lead time and more. Laser Powder Bed Fusion (LPBF), in particular, consists in selective fusion of regions of a powder bed by means of a fast-scanning high-power laser beam. It is notably characterized by very high solidification and cooling rates, as high as 10^5^–10^6^ °C/s, as well as repeated and sharp heating-and-cooling cycles as the laser beam travels over the powder bed and the consolidated parts according to the selected scanning strategy [1,2,3,4,5,6]. These unique characteristics, as opposed to conventional manufacturing, are responsible for the peculiar microstructure of LPBFed parts. This microstructure can be effectively controlled, to some extent, by varying the numerous process parameters. However, this severe thermal cycling also leads to stresses sometimes approaching or even exceeding the elastic limit of the materials associated with the build-up of anisotropic residual stresses. As a result, these high levels of residual stress may cause parts’ distortion and/or cracking thus significantly affecting mechanical properties (fatigue in particular). The reader is conveniently invited to refer to the excellent review recently published by Bartlett and Li [7] addressing residual stresses in metal powder bed fusion. While processing parameters can also be optimized with regards to their significant effect on residual stress buildup, post-processing stress relieving heat treatments at elevated temperatures to be applied shortly after manufacturing are often advised in order to release these stresses to some extent.

In previous studies, the effects of different heat treatments on microstructure and associated tensile properties of Alloy 625 produced by LPBF were investigated [8,9,10]. Alloy 625 is a popular Nb-rich solid-solution strengthened Ni-based superalloy characterized by attractive high temperature resistance for applications in aerospace and land-based gas turbines in particular [11,12,13,14]. In previous works, we found that the microstructure fully recrystallized upon solution treatment above 1150 °C [8,9,10]. This is remarkable as for Alloy 625, and most polycrystalline materials for that matter, recrystallization is usually achieved by application of external stress such as cold working followed by heating above the recrystallization temperature (which depends on the amount of cold work). Dynamic recrystallization is also possible by simultaneous application of stress and temperature during hot working. Recrystallization is a standard process of nucleation and growth of new strain-free and equiaxed grains from a severely strained microstructure, driven by the difference in internal energy (i.e., strain energy) between the strained and recrystallized material [15]. This suggests that strain energy built up in the as-built material produced by LPBF was high enough to lead to full recrystallization during solution treatment. A large number of authors also reported similar recrystallization after solution treatment of metals produced by LPBF [16,17,18,19,20,21,22,23,24,25,26,27], discussed later in Section 4.1. There exist several methods commonly utilized to evaluate stress levels within materials including the hole-drilling method, the contour method and diffraction methods such as X-ray Diffraction (XRD) and Neutron Diffraction (ND) [7]. While these methods provide some extent of quantitative characteristics of stress, their protocols may be challenging.

The as-built microstructure of Alloy 625 produced by LPBF is virtually always characterized by strong texture, large anisotropy, inhomogeneous solute distribution, a very fine dendritic structure, and a very high dislocations density primarily within the interdendritic regions [6,8,9,10,28,29,30,31]. The very high dislocations density, in particular, is the result of the peculiar solidification and thermal history inherited from LPBF, thus highlighting the high degree of strain energy previously mentioned. Perhaps naively, the assumption is that the higher the strain levels and associated residual stress, the higher the dislocations density and associated sub-micrometer deviation from perfect crystalline ordering. For this reason, electron microscopy methods such as electron backscattered diffraction (EBSD), which allow us to evaluate misorientation within crystals through Orientation Imaging Microscopy (OIM), seem appropriate to “visualize” strain levels. The present study uses EBSD to evaluate the effects of different 1 h–hold heat treatments at the standard temperatures of solutionizing (1150 °C), annealing (980 °C), stress-relieve (870 °C), and at an experimental low-temperature stress-relieve (650 °C). Microstructures and misorientation levels are studied to discuss the role of heat treatments and the suitability of EBSD techniques to assess residual stress in metals produced by LPBF.

## 2. Materials and Methods 

### 2.1. Raw Materials and LPBF Processing

Gas-atomized pre-alloyed Alloy 625 powder suitable for LPBF production was provided by the machine manufacturer EOS GmbH (Munich, Germany). The element composition of the powder given by the supplier and verified by Inductively Coupled Plasma (ICP) and Infrared absorption (IR) analyses is reported in Table 1. Most particles were spherical, with a typical Gaussian size distribution measured between *d*_10_ = 16 µm and *d*_90_ = 48 µm. Samples were produced with a commercial EOSINT M270 Dual Mode machine (EOS GmbH, Munich, Germany). The scanning strategy used stripes of 5 mm overlapping by 0.12 mm and a laser scanning direction rotated by 67° between subsequent layers. Optimized parameter conditions for Alloy 625 were utilized (laser power P = 195 W, scanning speed v = 1200 mm/s, hatching distance h = 90 µm and layer thickness t = 20 µm). These conditions are the result of an optimization campaign leading to the production of specimens with a residual porosity less than 0.1% [30]. Thereby, 100 mm–long cylindrical specimens with a diameter of 25 mm were produced, vertically, for characterization and subsequent heat treatments.

### 2.2. Heat Treatments

**Figure 1:**
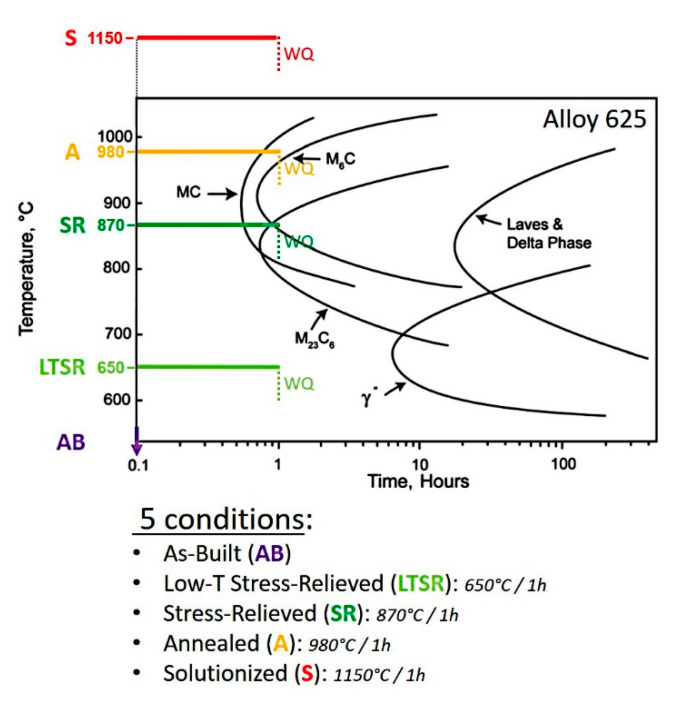
Schematic diagram of the different heat treatments superimposed on the conventional Time–Temperature Transformation (TTT) diagram for Alloy 25 [12].

The effects of different 1 h–hold heat treatments at different temperatures on the microstructure were evaluated by microscopy. The Alloy 625 standard temperatures of solutionizing (1150 °C, S), annealing (980 °C, A) and stress-relieve (870 °C, SR) were considered. In addition, an experimental low-temperature stress-relieve temperature of 650 °C (LTSR) was evaluated. A schematic diagram of the different heat treatments in correspondence with the conventional Time–Temperature Transformation (TTT) diagram for Alloy 625 is shown in Figure 1. The TTT diagram in Figure 1 is generally accepted for the conventional wrought Alloy 625 [12,14]. It is, nevertheless, reasonable to assume that the effective TTT diagram for Alloy 625 produced by LPBF may be different due to its specific nature. Lindwall et al. [29,32], for example, modeled segregations and explained, in particular, the more rapid precipitation kinetics of the δ-phase with respect to wrought Alloy 625.

All heat treatments were carried out in a muffle furnace with a ±5 °C precision, equipped with a K-type thermocouple located close to the sample. Each specimen was eventually quenched in water to preserve microstructure. It should be noted that water quenching, due to rapid cooling, induces thermal contraction residual stress particularly significant at the periphery of cylindrical specimens, cooling first and rapidly. This quenching stress prevents the use of XRD for assessing LPBF process residual stress levels on the surface of specimens. 

### 2.3. Microstructure Characterization and Residual Strain Levels

**Figure 2:**
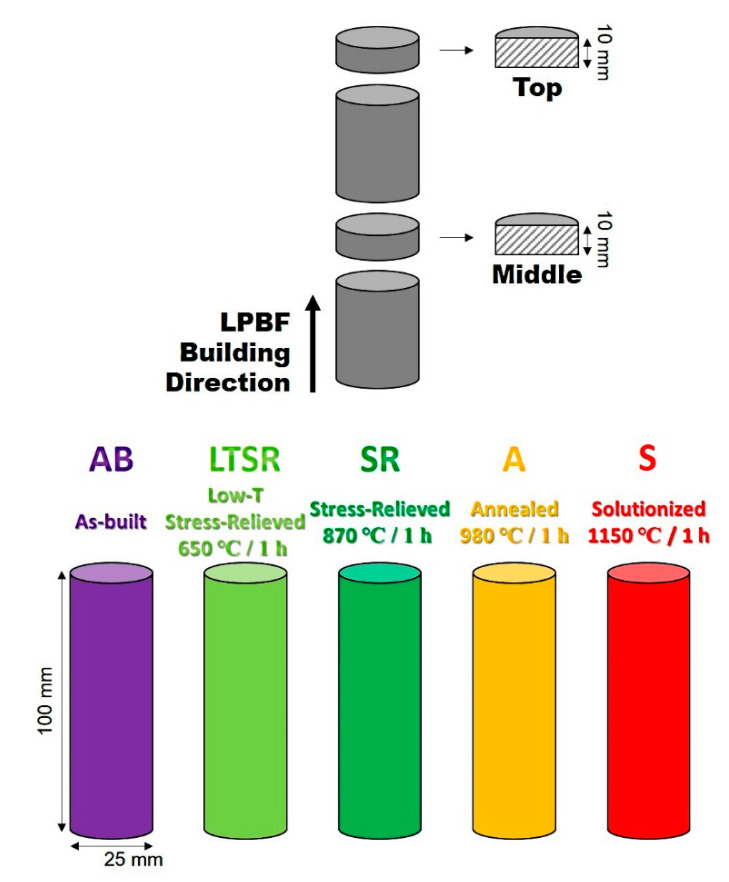
Schematic diagram of the specimens and 5 different conditions.

Specimens for microstructure analyses were cautiously sectioned, hot mounted in phenolic resin and carefully ground with SiC abrasive papers, followed by sequential polishing with diamond paste down to 1 μm. The microstructure was revealed by means of a 100 mL HCl + 0.5 g CrO_3_ etching solution and investigated by optical microscopy (OM, OLYMPUS BX51M, Tokyo, Japan) and scanning electron microscopy (SEM, JSM-6510, JEOL, Tokyo, Japan). Specimens for transmission electron microscopy (TEM, field-emission type JEM-2100F operating at 200 kV, JEOL, Tokyo, Japan) were extracted from the middle of specimens. Thin foils were prepared by fine grinding and polishing, to a thickness of approximately 200 µm, punching of 3 mm diameter discs and electropolishing to perforation with a 200 mL perchloric acid + 800 mL methanol solution at −26~−28 °C, using a jet polisher (TenuPol-5, Struers, Copenhagen, Denmark) operating at 50 mA and 25 V. Electron Backscattering Diffraction (EBSD) with a field-emission scanning electron microscope (FE-SEM, MIRA-II, TESCAN, Brno, Czech Republic) was carried out. To avoid artifacts from metallographic preparation, the final 1 µm polishing step was executed, using a colloidal silica suspension, and no etching solution was used. All five cylindrical bars were sectioned to extract approximately 1 cm^2^ specimens in the middle and at the top for observation in the cross-section parallel to the building direction. A schematic diagram of the samples is given in Figure 2. 

## 3. Results

### 3.1. Microstructure

**Figure 3:**
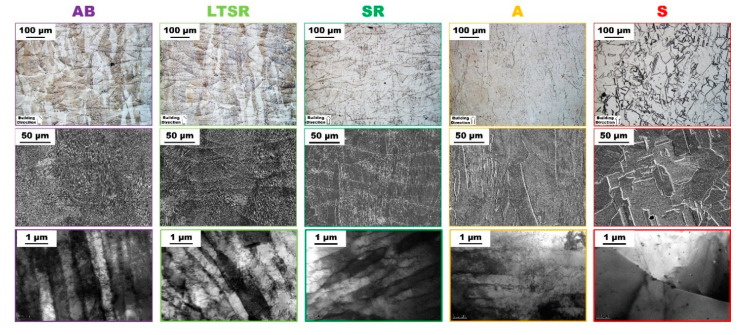
Optical (top), scanning electron (middle) and transmission electron (bottom) micrographs of as-built (AB), low-temperature stress-relieved (LTSR), stress-relieved (SR), annealed (A) and solutionized (S) in the middle of Alloy 625 specimens produced by LPBF. The TEM micrograph for AB (bottom-left) was reproduced with permission from [8].

The microstructure of the Alloy 625 produced by LPBF as-built and after the four isothermal heat treatments was observed at different length scale. Optical microscopy (OM) allows evidencing the typical processing features proper to the LPBF process, such as melt pools, as well as the grain structure. Scanning electron microscopy (SEM) highlights the cellular/dendritic structure and segregation. Transmission electron microscopy (TEM) reveals the finer details, in particular, subgrain structure and dislocations. Figure 3 shows the corresponding microstructures in the middle of the cylindrical specimens. 

The microstructure of the as-built material (AB in Figure 3) was characterized by a very fine cellular/dendritic structure with grains intercepting several melt pools elongated along the building direction. Melt pools are clearly visible on the representative OM and SEM micrographs, characterized by the so-called fish-scale structure. The rapidly solidified AB microstructure is also characterized by inhomogeneous distribution of the constituting elements of Alloy 625, illustrated in particular by different contrasts in the SEM micrograph. The finer features of the typical AB microstructure were evidenced by the high-magnification TEM micrographs in Figure 3. At this scale, the microstructure exhibited a very fine cellular/dendritic structure, high dislocations density particularly within interdendritic areas and the presence of very fine Nb-carbides with a size ≤50 nm. The high density of dislocations is indicative of accommodation for residual thermal stress due to the rapid cooling and heating cycles inherent to the LPBF process and therefore illustrates the high levels of strain energy accumulated within the material. This peculiar microstructure is typical of alloys produced by AM powder bed fusion processes such as LPBF [6,8,9,10,28,29,30,31]. 

The microstructure of the low-temperature stress-relieved specimen (650 °C, LTSR in Figure 3) was nearly identical to that of the as-built material. Melt pools, elongated grains, segregation, cellular/dendritic structure, and high dislocations density were clearly identified. In a previous study [8], hardness values varied after aging between 600 and 700 °C for 2 h. Although no clear evidence emerged from TEM observations, the formation of phases such as γ’’ or δ cannot be excluded at this temperature due to faster kinetics of precipitation in AM alloys [29,32]. 

After one hour at the standard temperature of stress-relieve 870 °C (SR in Figure 3), the microstructure was also similar to that of the reference AB alloy. There was no sign of recrystallization, melt pools were still clearly visible and the fine dendritic structure was preserved. The dislocations density of SR in Figure 3 appears slightly lower compared to AB; however, drawing a conclusion is difficult due to the very small volume of analysis allowed by TEM (~100 μm^3^). Significant precipitation of the δ-phase has been detected in these conditions similarly to a previous research [10], which is consistent with the more rapid precipitation kinetics of the δ-phase as a result of segregation mentioned earlier. δ-phase formation under similar thermal exposure (from 700 to 900 °C) for prolonged times (200 h) was also reported, revealing that segregations tend to limit its growth [33].

After annealing for 1 h at 980 °C (A in Figure 3), the microstructure exhibited clear differences with that of AB. While virtually no signs of recrystallization were presently observed (OM and SEM micrographs in Figure 3), melt pools were barely visible by OM and clearly less pronounced by SEM. The absence of melt pool contours had also been observed in a previous research and attributed to homogenization [10]. It is believed that melt pools’ boundaries are revealed by chemical etching due to significantly different concentration of solute elements as a result of the solidification sequence. Occasional signs of recrystallization were also observed in Reference [10], suggesting that recrystallization may be promoted at this temperature upon longer exposure. At lower scale, while a fine dendritic structure could be observed in some location, homogenization was clear, and the dendritic structure disappeared otherwise. The TEM micrograph (A in Figure 3) highlights the relatively high dislocations density remaining in spite of the heat treatment, as well as the disappearance of the heavily segregated and strained interdendritic regions. The presence of fine nanometric Nb-carbides was also evidenced; however, it was not clear whether nucleation or growth dominated from the AB state.

On the contrary, the microstructure in the middle of the solutionized alloy (S in Figure 3) exhibited fully recrystallized equiaxed grains with an approximate grain size of 80 µm and significant amount of twinning (annealing twins). For this Alloy 625 produced by LPBF, the formation of equiaxed grains and annealing twins typically occur under high temperatures [8,9,10,27]. In this case, the S state present slightly smaller equiaxed grains with respect to the solutionized version performed with a holding time of 2 h mainly around 90 µm also with a fraction of smaller grains around 10 µm, as reported in a previous work [8]. A large amount of fine globular precipitates was observed all over the material. These were identified as Nb-rich MC carbides. Similar NbC precipitates have been reported in Alloy 625 produced by SLM and subjected to solution treatments [8,9,10,28,34]. The representative TEM micrograph of the solutionized alloy highlights the recrystallized structure with only a low amount of dislocations.

### 3.2. Electron Backscattered Diffraction Analysis

**Figure 4:**
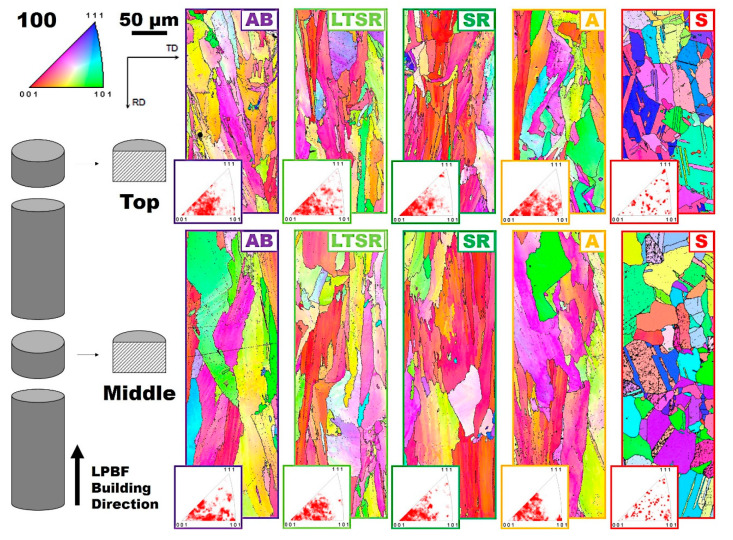
Inverse Pole Figure (IPF) maps at the top and in the middle of as-built (AB), low-temperature stress-relieved (LTSR), stress-relieved (SR), annealed (A) and solutionized (S) Alloy 625 specimens produced by LPBF.

As mentioned in introduction, electron backscattered diffraction (EBSD) analyses by means of scanning electron microscopy allows understanding crystallographic aspects of microstructure, such as the crystallographic orientation. As a consequence, it allows mapping and quantifying, to some extent, ordering deviations within crystals, more precisely orientation deviations from neighboring points within grains. Figure 4 shows the Inverse Pole Figure (IPF) maps recorded at the top and in the middle of all five Alloy 625 specimens produced by LPBF and subjected to the heat treatments considered (see Figure 1). IPF data show the variations with crystallographic orientations and therefore is a valuable approach to evaluate texture. It is clear in Figure 4 that, while only the alloy submitted to solutionizing (S in Figure 4) exhibited a fully recrystallized microstructure with randomly oriented equiaxed grains and significant twinning (annealing twins), the alloys heat treated at lower temperatures (A, SR and LTSR in Figure 4) had elongated grains along the building direction oriented preferentially along <001>, similar to the as-built material (AB in Figure 4). Inserts in Figure 4 clearly highlight texture. This indicated, in particular, that 1 h at the relatively high temperature of annealing (980 °C) was not sufficient to promote recrystallization of the as-built Alloy 625 produced by LPBF. Similar results were obtained in a recent study [10]. There seemed to be little significant difference between the middle and the top of specimens besides a slightly lower grain size at the latter, processed last.

**Figure 5:**
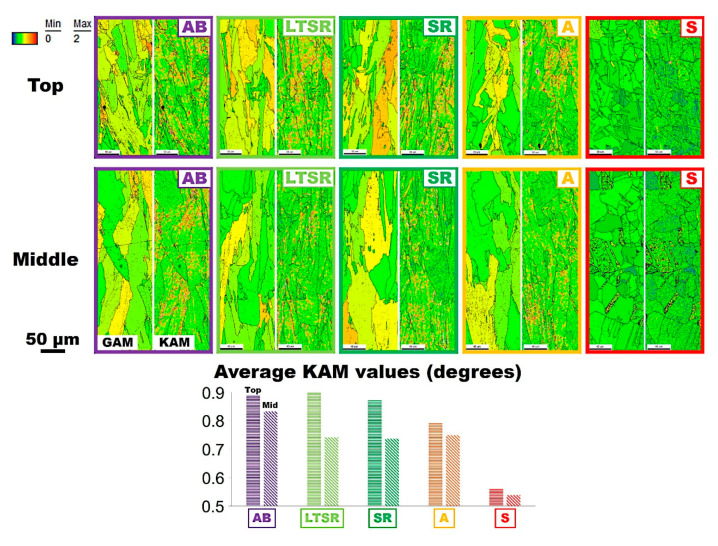
Grain (left) and kernel (right) average misorientation maps at the top and in the middle of as-built (AB), low-temperature stress-relieved (LTSR), stress-relieved (SR), annealed (A) and solutionized (S) Alloy 625 specimens produced by LPBF. Average values of kernel average misorientation (KAM) are also plotted.

The peculiar processing conditions of innovative LPBF technologies induce steep thermal cycles, including partial remelting of the underlying consolidated material. This, as mentioned previously, generates large stresses in the material due to rapid thermal expansion and contraction, which causes strain [7]. This plastic residual strain in turns manifests within relief of the distortion in the crystal lattice by the formation of dislocations. An excellent review by Wright et al. [35] explores the use of EBSD analyses for evaluating strain levels in materials. It suggests, in particular, that, while EBSD is limited to qualitative analyses, it is a powerful means for visualization of strain, using orientation imaging microscopy (OIM) maps since dislocations and dislocations density describe plastic strain levels. As described by Wright et al. [35], the residual strain caused by dislocations forming in the material is apparent as local lattice orientation variations, which therefore indicate strain distribution within the material. Figure 5 compares grain average misorientation (GAM, left) and kernel average misorientation (KAM, right) maps at the top and in the middle of all five Alloy 625 specimens. GAM is the average misorientation between each neighboring pair of measurement points within the grain. KAM is the average misorientation between all neighboring points within a defined kernel.

In Figure 5, the as-built material (AB in Figure 5) exhibited severe residual strain levels manifested by large degree of local misorientation and illustrated by lighter yellow and orange regions in both GAM and KAM maps. Average KAM values in Figure 5, to some extent, representing comparatively the level of strain energy within the considered region, indicated values well over 0.8 degrees. This is representative of heavily stressed as-built materials produced by LPBF, as mentioned previously. There was a slightly higher level of misorientation detected at the top of the as-built bar. While significance is doubtful due to statistical considerations and the relatively small area considered by EBSD (approximately 43,000 μm^2^), similar results for the other conditions bring confidence. On the contrary, after one hour at the solutionizing temperature of 1150 °C (S in Figure 5), strain levels were much lower (illustrated qualitatively by green regions) with average KAM values approaching 0.5 degrees. The full recrystallization process is likely responsible for releasing a large amount of strain in the material, in particular by accommodating both statistically stored dislocations (SSDs) and geometrically necessary dislocations (GNDs). GAM and KAM maps showed similar features at the top and in the middle of the cylindrical specimen. 

From Figure 5, the effects of heat treatments at lower temperatures (A, SR and LTSR in Figure 5) were more puzzling. As first consideration, there are notable differences between the top and the middle of specimens. This is highlighted by the average KAM values bar chart, at the bottom, in Figure 5. It also is intuitively observed on both GAM and KAM maps comparing the top and middle regions. The top of the samples, regardless of the condition of the specimen, exhibited higher strain levels than the middle. The effect of heat treatments at temperatures below recrystallization seems to be more impactful in the middle than at the top of the specimens. 

With considerations to the middle of the cylindrical specimens, the impact of temperature was surprisingly not clear. All three unrecrystallized materials exhibited similar strain levels with average KAM values around 0.75 degrees, in spite of significantly different heat-treatment temperatures. Differently, EBSD results from the top of the specimens subjected to different temperatures suggested, as one could anticipate, that higher temperature promotes stress-relieving to the point of full recrystallization at least above 1150 °C. The materials subjected to the low-temperature stress-relieve heat treatment (LTSR in Figure 5) exhibited strain levels at the top similar to that of the as-built specimen. Likewise, while recorded values appeared slightly lower, strain levels at the top after the stress-relieve treatment (SR in Figure 5) were similar to that of the as-built specimen. After heat treatment at the higher temperature of annealing (A in Figure 5), strain levels at the top were clearly lower than for the as-built material, with average KAM values below 0.8 degrees. With comparison to the fully recrystallized materials after solutionizing (S in Figure 5), residual stain levels were still clearly higher for A, consistent with the fact that recrystallization is most effective for relieving stress.

## 4. Discussion

### 4.1. Recrystallization

**Figure 6:**
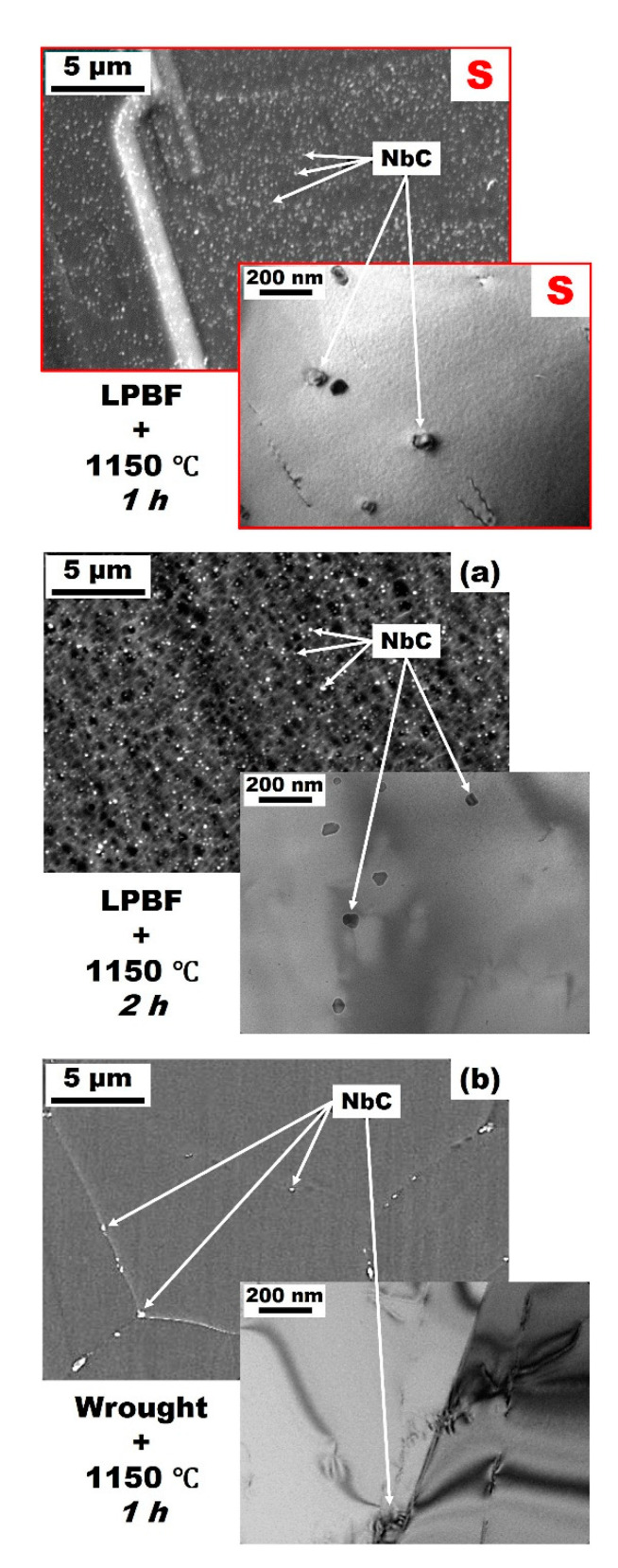
SEM and TEM micrographs of Alloy 625 produced by LPBF and submitted to 1 h–hold (S) and 2 h–hold (**a**) [8] solution treatment at 1150 ℃; (**b**) conventional wrought and solutionized at 1150 ℃ for 1 h Alloy 625.

The popular Alloy 625 was produced by LPBF and subjected to 1 h–hold heat treatments at different temperatures. Perhaps the most obvious consideration that can be drawn from the results is that recrystallization was complete after heat treatment at 1150 °C (S in Figure 1) for 1 h. This was particularly clear by microscopy (S in Figure 3) and from IPF data (S in Figure 4): The microstructure after S consisted in fine strain-free equiaxed grains with random orientation. This recrystallization accommodates the heavily stressed as-built microstructure to resemble the conventional recrystallized microstructure of its wrought counterpart. Such observation was already mentioned in previous studies where full recrystallization of Alloy 625 similarly produced by SLM was observed after solution treatment at the same temperature of 1150 °C for 2 h [8,9,10]. The present study confirms that 1 h at 1150 °C is sufficient to promote full recrystallization. Figure 6 shows representative SEM and TEM micrographs of Alloy 625 produced by LPBF and subjected to either the present 1 h–hold at 1150 °C (S in Figure 6) or a 2 h–hold at 1150 °C (Figure 6a), [8]). In addition, representative SEM and TEM micrographs of a conventional wrought Alloy 625 [9] subjected to a similar 1 h–hold at 1150 °C were included in Figure 6b for comparison. 

Comparing the effect of the holding time at temperature between 1 and 2 h (S and a in Figure 6, respectively), there seemed to be no significant difference. As mentioned previously, the alloys produced by LPBF and subjected to solution treatments clearly exhibited a large number of fine globular precipitates, identified as Nb-rich MC carbides, distributed within the γ-matrix. Although it has not been appropriately quantified due to length scale considerations, the size distribution of these carbides was slightly larger after 2 h than after only 1 h. It could also be noted that grain size was also slightly higher after 2 h (approximate maximum grain size of 90 μm), compared to 1 h–hold (approximate maximum grain size of 80 μm). These were significantly smaller than the average grain size of a wrought and similarly solutionized alloy at 1150 °C for 1 h (~130 μm), as discussed in Reference [9]. It could be noted here that much smaller grain size, critical for its contribution to strength, is usually observed for conventional wrought Alloy 625 (for example hot-rolled, in absence of solutionizing), with approximate grain size as low as 15 μm on average [11,13,36,37]. 

The most significant difference between Alloy 625 produced by LPBF and its conventional solutionized wrought counterpart in Figure 6b was the significant presence of these Nb-rich MC carbides. While the wrought material exhibited only a small amount of relatively large solidification MC precipitates despite the solution treatment (Figure 6b), the volume fraction in the material produced by LPBF was clearly much higher (S and a in Figure 6). Briefly mentioned earlier in Section 3.1, the large presence of very fine Nb-carbides with a size ≤50 nm was systematically observed within the as-built material [8,9,10]. This contrasts with much larger primary Nb-rich MC carbides precipitated during solidification and often distributed along the rolling direction in conventional wrought Alloy 625 [11,13,36,37]. As a result, it is reasonable to assume that the very fine Nb-carbides of the as-built material experienced significant growth during the solution treatment at the relatively low temperature of 1150 °C. This is consistent with the NbC start precipitation temperature for Alloy 625 reported at 1250 °C by Zhao et al. [38], which contrasts with the TTT diagram for Alloy 625 given in Figure 1 [12,14]. The pinning effect of this fine precipitation may well be an important factor for the relatively low grain coarsening rate during solution treatment with grains only growing by about 10 μm in diameter as a result of an additional one-hour hold at temperature. The resulting microstructure after solution treatment led to tensile properties at room temperature in terms of strength and ductility favorably comparable to conventional wrought Alloy 625 [8,9,10].

Recrystallization of metals produced by LPBF when subjected to high-temperature solution heat treatments has been reported for a wide variety of materials. For example, recrystallization was reported for a 316L stainless steel produced by LPBF and exposed to solution treatment at 1200 °C for 2 h [16,17]. Recrystallization of a similar alloy 316L produced by LPBF and subjected to only 1100 °C for 10 min was only observed after laser shot peening [18]. Recrystallization was, in particular, correlated to higher local misorientation level after laser shot peening. The recrystallization temperature for another LPBF alloy 316L, defined as the temperature at which recrystallization reaches 50% after 1 h, was evaluated at 1100 °C [19]. An LPBF superalloy IN718 exhibited fully recrystallized grains after solutionizing at 1150 °C for 2 h, while homogenization at the lower temperature of 1065 °C for 1.5 h was found to be insufficient [20]. In another study, this alloy IN718 produced by LPBF exhibited a much higher of volume fraction of recrystallized grains after a simulated HIP treatment, under normal pressure, at 1160 °C, for 4 h, than after an initial stress relief annealing treatment at 982 °C for 0.5 h, followed by HIP (1163 °C, 4 h, 100 MPa pressure in Ar) [21]. This alloy, produced by an alternative yet similar PBF process called laser solid form, also experienced recrystallization after being subjected to a first solutionizing step at 1100 °C for 1.5 h, with the authors associating inhomogeneous distribution of recrystallized grains to uneven distribution of residual thermal stress [22]. Superalloy IN939 produced by LPBF and exposed to 1160 °C for 4 h also exhibited recrystallized grains [23]. Recrystallization and annealing twins were also reported for a high-γ’ PM superalloy produced by LPBF and subjected to solutionizing at 1180 °C for 1 h [24]. Recrystallization after LPBF and solution/annealing heat treatments at sufficiently high temperature was also reported for pure iron [25], titanium [26] and more. 

This brief and non-exhaustive review of the literature suggests, in particular, that the residual stress levels built-up during the LPBF process due to repeated and sharp heating and cooling cycles promote recrystallization in a large number of metals during solution treatment at a temperature above the recrystallization temperature of the alloy. This recrystallization temperature usually depends on the amount of deformation (for example, cold work)—more precisely, strain energy— which the alloy had been subjected to. In the case of the LPBF process, it suggests that the recrystallization temperature is a function of the strain levels generated during production. While these strain levels can be controlled, to some extent, by careful optimization of the processing parameters, it seems that strain in alloys produced by LPBF approach those of conventionally wrought materials (that is corresponding to about 20% to 30% deformation). 

### 4.2. Inhomogeneous Distribution of Strain and Role of Heat Treatment

The results presented in Section 3 suggested that residual strain levels are uneven within materials produced by LPBF. This was clear from the distribution of strain levels (local misorientation levels) in KAM maps in Figure 5. This can be explained, in particular, by the nature of heat transfers during production, which depends on the building strategy (scanning path strategy, layer rotation, scanning speed, overlapping, laser power, etc.). A simplistic view of residual stress formation during LPBF would be that, due to rapid scanning of the heat source, the abrupt thermal expansion as it approaches and thermal contraction as it moves away of a given location generate large thermal stresses accommodated by the formation of dislocations. Transformation stress for some alloys, such as some steels or titanium alloys, for example, may also be significant. It should be also understood that thermal stresses generated by the moving heat source also significantly influence the distribution of stress (or strain) along the building direction. As the production by LPBF proceeds with subsequent layers, the underlying material already consolidated into shape does receive heat in quantity inversely proportional to the distance from the heat source. The heat energy provided during the process is often given as the energy density *E*_d_ in J.mm-3, which is a function of the principal process parameters [30]. Not relevant in the present study, due to consistent cylindrical shape of the as-built specimens, the geometry of the built parts plays an important role, as well, particularly when considering that heat conduction and convection varies between powders and dense solids. 

This therefore suggests, in simple terms, that not only stress levels and accordingly strain levels vary along the height of as-built part, but also the nature of the residual stress varies. In their review, Bartlett and Li [7] reported for example that the top of as-built parts are usually in tension, while the middle portions are in compression. Moreover, as the process progresses, underlying consolidated material undergoes some extent of heat treatment, by diffusion from the heat source through the part, in spite of severe dynamic considerations. It seems therefore reasonable to assume some degrees of stress relieving, considering, in particular, the mechanism of dislocations recovery, as well as homogenization despite limited diffusion due to abrupt heating and cooling together with relatively short effective time at temperature. Additional evidence relies on the slightly larger grain size in the middle compared to the top, suggesting grain growth was lightly promoted during production. This is consistent, in particular, with the slight difference in strain levels between the top and the middle section of as-built materials (AB in Figure 5). It should however be noted that the residual strain levels “visualized” by EBSD data consist of local misorientation at the microscale level caused, in particular, by the formation of dislocations to accommodate these thermal stresses. As such, OIM data, in particular, seem appropriate to evaluate qualitatively the extent of microscopic strain energy stored within the material due to thermal stresses generated during the process. However, it is in no way an actual measure of residual stress. This is clear, as from the results in Figure 5 the tensile or compressive nature of the stored residual stress is unknown. In simple words, OIM data seem to allow us to visualize the local extent of stress to which the material was subjected to through recording of misorientation levels caused by the formation of dislocations to accommodate this stress. Lower levels of local misorientation or strain levels observed in the middle of the cylindrical specimens in Figure 5, indicating lower residual stress levels, are therefore coherent due to the aforementioned exposure to heat within the middle regions of parts. This is, however, not counting on the nature (compression or tension) of residual stress.

LPBF manufacturing is characterized by the sequential selective melting and solidification of very small volumes of material by the fast scanning laser heat source. This brief input of high-heat energy leads to abrupt local thermal cycles, which produces rapid thermal expansion and contraction generating large thermal stresses. The very rapid solidification and heat cycles sequence lead, in particular, to a very fine cellular/dendritic structure, fine segregation of constituting elements and very high dislocations density to accommodate rapid solidification and thermal stresses. OIM data obtained by EBSD allow us to record crystallographic misorientation levels (GAM and KAM data, for example, in Figure 5), caused, in particular, by the abovementioned dislocations. However, for appropriately good resolution FE-SEM EBSD measurements, the contribution of punctual crystalline defects (vacancies or more importantly alloying elements in solution) may well be significant. Since post-processing heat-treatment temperatures have a major effect on diffusion and solubility within the solid, OIM data might also return segregation/homogenization levels, which contribute to some extent to local strain energy levels.

**Figure 7:**
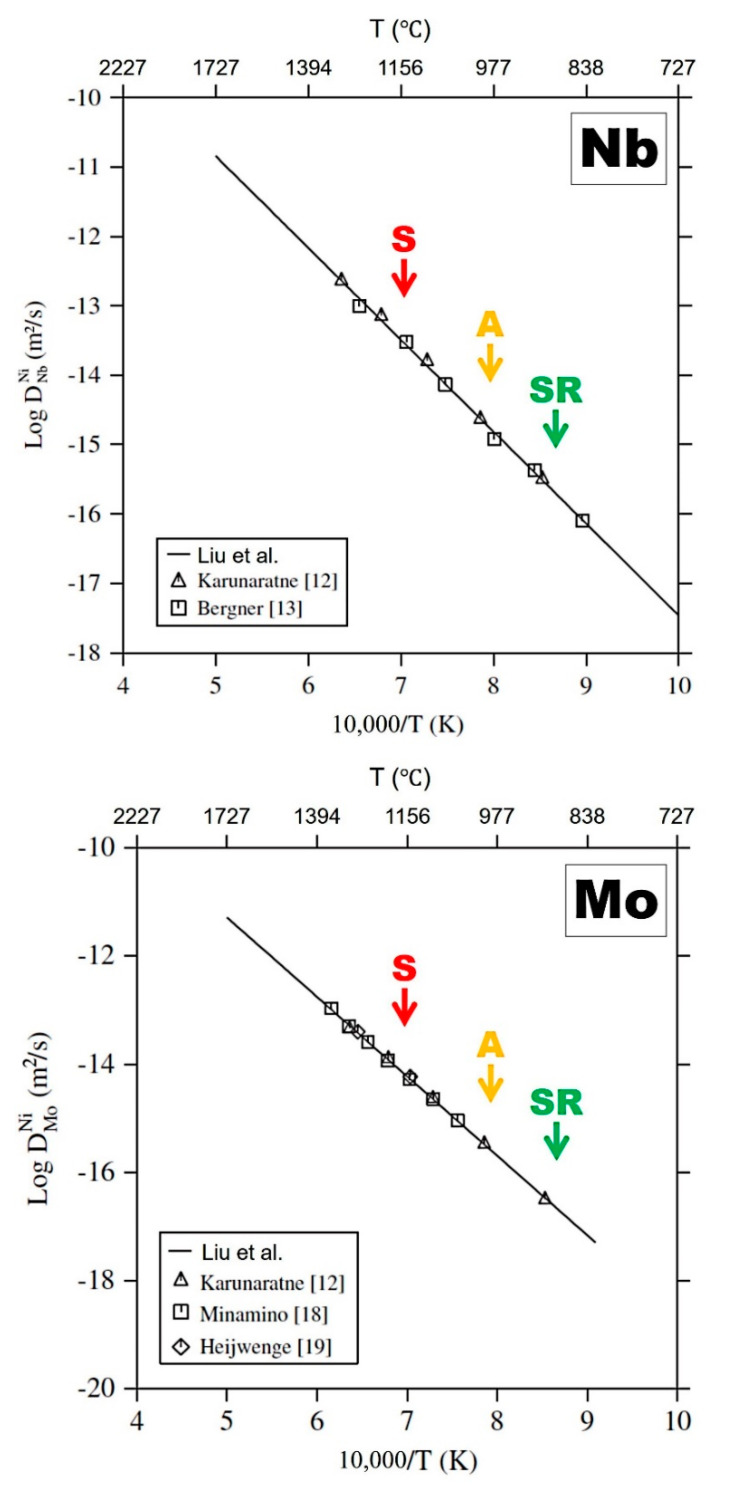
Diffusion coefficients into Ni of Nb and Mo as a function of temperature reported by Liu et al. [39].

OIM data obtained by EBSD, and local misorientation data, in particular, are sensitive to the presence of dislocations. However, it seems reasonable to assume that the presence of alloying elements in solution, in particular, large elements such as Nb and Mo, may generate local misorientation recorded by EBSD, providing sufficient resolution (i.e., high-resolution FE-SEM). Although it is difficult to confirm, local segregation of elements may influence, to a non-negligible extent, KAM data in particular. Assuming this conjecture is revealed correct, the effects of homogenization due to different heat-treatment temperatures should be taken into account. In a previous research study [10], XRD was used to measure lattice parameter average values of alloys under similar conditions, in order to define homogenization levels. It was found that increasing temperature promoted homogenization in LBPFed Alloy 625. Figure 7 shows the diffusion coefficients in Ni of Nb and Mo as reported by Liu et al. [39]. In Figure 7, the corresponding temperatures of the selected heat treatments (S, A and SR in Figure 1) were indicated. Obviously, diffusion of both Nb and Mo is significantly promoted as temperature increases. This therefore suggests that the extent of homogenization, larger after heat treatment at higher temperature, may contribute to the reduction of average KAM values observed in Figure 5. This also is consistent to the homogenization of the microstructure, melt pool boundaries and heavily segregated dendritic structures observed for A in Figure 3. 

## 5. Conclusions

The popular Alloy 625 was produced by LPBF and the as-built microstructure was compared to those obtained after 1 h–hold heat treatments, at the different standard temperatures of solutionizing (1150 °C), annealing (980 °C), stress-relieve (870 °C) and at an experimental low-temperature stress relieve (650 °C). The microstructure and its features were characterized at different length scale by microscopy, and EBSD techniques were used to evaluate local crystallographic misorientation levels associated with residual stress. OIM data obtained from EBSD allowed us to visualize and evaluate local misorientation levels and distribution, which correlated with residual stress formation. While EBSD does not return quantitative values of residual stress levels, it allows to investigate the amount and distribution of strain energy levels caused by crystallographic defects such as dislocations themselves produced to accommodate the large thermal stresses generated during LPBF. Above recrystallization at 1150 °C, a strain-free microstructure formed, and most residual stress was relieved. Below recrystallization, increasing the heat treatments’ temperature promoted dislocation recovery and homogenization, which clearly contributed to release stress at the top of the cylindrical specimens. However, the contribution of higher temperatures was less clear in the middle of specimens. 

## Figures and Tables

**Table 1:** Chemical composition of the Alloy 625 powder in weight percent.

Composition	Ni	Cr	Mo	Fe	Nb	Co	Si	Ti	Al	C
Supplier	≥58.0	20–23	8–10	≤5	3.15–4.15	≤1.0	≤0.5	≤0.4	≤0.4	≤0.1
ICP/IR	Bal.	22.8	8.1	0.43	3.66	0.17	0.1	0.17	<0.01	0.013
